# An interpretable framework to identify responsive subgroups from clinical trials regarding treatment effects: Application to treatment of intracerebral hemorrhage

**DOI:** 10.1371/journal.pdig.0000493

**Published:** 2024-05-07

**Authors:** Yaobin Ling, Muhammad Bilal Tariq, Kaichen Tang, Jaroslaw Aronowski, Yang Fann, Sean I. Savitz, Xiaoqian Jiang, Yejin Kim

**Affiliations:** 1 D.Bradley Mc.Williams School of Biomedical Informatics, UTHealth at Houston, Houston, Texas, United States of America; 2 Department of Neurology, University of California, Los Angeles, Los Angeles, California, United States of America; 3 Institute for Stroke and Cerebrovascular Disease, UTHealth at Houston, Houston, Texas, United States of America; 4 Intramural Research Program, National Institute of Neurological Disorders and Stroke, National Institutes of Health, Bethesda, Maryland, United States of America; Reader in Forensic Intelligent Data Analysis, UNITED KINGDOM

## Abstract

Randomized Clinical trials (RCT) suffer from a high failure rate which could be caused by heterogeneous responses to treatment. Despite many models being developed to estimate heterogeneous treatment effects (HTE), there remains a lack of interpretable methods to identify responsive subgroups. This work aims to develop a framework to identify subgroups based on treatment effects that prioritize model interpretability. The proposed framework leverages an ensemble uplift tree method to generate descriptive decision rules that separate samples given estimated responses to the treatment. Subsequently, we select a complementary set of these decision rules and rank them using a sparse linear model. To address the trial’s limited sample size problem, we proposed a data augmentation strategy by borrowing control patients from external studies and generating synthetic data. We apply the proposed framework to a failed randomized clinical trial for investigating an intracerebral hemorrhage therapy plan. The Qini-scores show that the proposed data augmentation strategy plan can boost the model’s performance and the framework achieves greater interpretability by selecting complementary descriptive rules without compromising estimation quality. Our model derives clinically meaningful subgroups. Specifically, we find those patients with Diastolic Blood Pressure≥70 mm hg and Systolic Blood Pressure<215 mm hg benefit more from intensive blood pressure reduction therapy. The proposed interpretable HTE analysis framework offers a promising potential for extracting meaningful insight from RCTs with neutral treatment effects. By identifying responsive subgroups, our framework can contribute to developing personalized treatment strategies for patients more efficiently.

## Author summary

In our research, we tackle a common problem in medical studies where treatments often don’t work the same for everyone, leading to many failed experiments. Imagine trying to find a key that not only fits a specific lock but also works better for certain types of locks. That’s what we aimed to do for a serious brain condition called intracerebral hemorrhage (ICH), which currently has no widely accepted treatment. We developed a new approach that helps us look back at unsuccessful trials and identify specific groups of patients who might benefit from a treatment that was deemed ineffective on a broader scale. To make our method even stronger, we used additional data from past studies and created artificial data with the help of cutting-edge computer models. This way, we make our framework work better in real-world scenarios. Our findings led to the discovery of important patterns that doctors could use to tailor treatments more effectively for individuals suffering from ICH. By doing so, we hope to pave the way for more personalized and successful treatment plans in the future, offering new hope for patients facing this life-threatening condition.

## Introduction

The success rate of clinical trials was estimated to be only 13.8%, [1], and an investigation of 640 Phase III trials found that around 57% of them failed due to inadequate efficacy. [2] The success rate is much lower for some diseases without disease-modifying therapies. For example, intracerebral hemorrhage (ICH) is a devastating form of stroke, with the highest mortality rate of all stroke subtypes and severe disability affecting ICH survivors. [3] Many efforts have been devoted to identifying effective therapies to help patients recover from the disease. [4, 5] Several Phase II and III trials for developing therapies have been conducted, such as ATACH2, [6] MISTIE III, [7, 8] and i-DEF, [9] but none have shown significant positive effects on primary endpoints in improving outcomes. While some of these studies have been neutral for the enrolled population, several indirect pieces of evidence support nontrivial treatment effects in some patient subpopulations. [10–13] Recently, an international multicenter Phase III trial evaluated a care bundle protocol to improve a patient’s functional outcome after an acute ICH disease. It showed that patients’ modified Rankin Scale (mRS) scores were improved with statistical significance by controlling multiple physiological measurements. [14]

As we can learn from some trials, treatment effects on individuals vary by many factors and combinations. For those failed trials, researchers believe the crude enrollment criteria to select patients might have overlooked patient heterogeneity and obscured their outcomes. [15] To identify patients who can benefit from the target treatment, earlier studies stratified the population by pre-specified subgroups, but they did not identify promising candidates. Testing hypotheses on manually selected stratification of one or two confounders is like finding a needle in a haystack. It might also suffer from oversimplifying intervention’s heterogeneous and nonlinear causal effects on primary outcomes.

Several data-driven approaches to discovering subgroups in terms of heterogeneous treatment effects (HTE) have been studied. Recursive partitioning methods, such as causal trees, were used to group patients by splitting subjects based on conditions that maximize separations; for details, see review papers. [16, 17] Linear regression was also used to investigate the heterogeneity in treatment effects while interpreting the covariates’ importance as a subgroup analysis. [18, 19] Recently, with the advance of various machine learning, the “digital twin” approach, which builds a supervised model to regress to factual or counterfactual outcomes, has been proposed, such as meta learners, [20] covariates shift, [21] and counterfactual regression; [22] see the review for methodology details. [23] These methods are mainly for predicting HTE but do not provide subgroups of patients with similar HTE.

**Fig 1:**
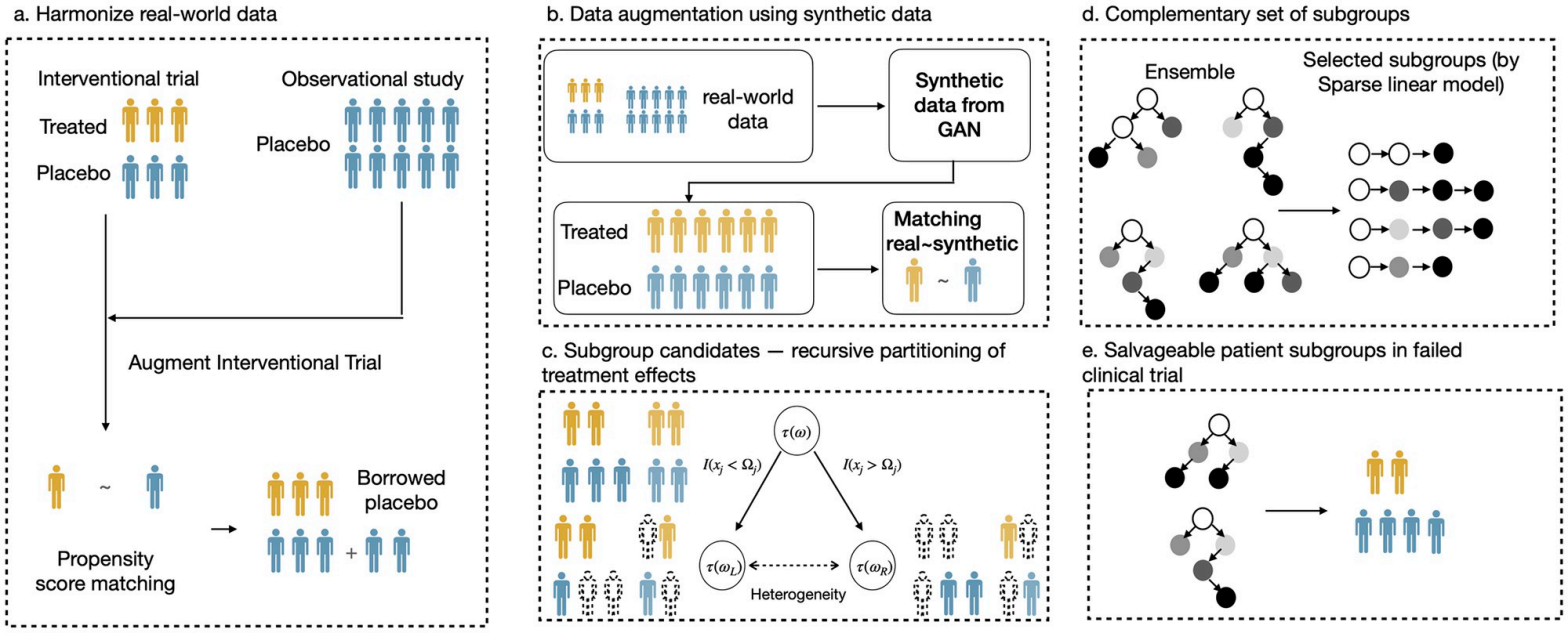
Framework overview. We first integrate individual-level data from an interventional trial and observational trial to increase sample size while maintaining the balance of confounders between treatment and placebo arms (Fig 1a). We then built a generative model to generate synthetic data that are like the real data and have similar confounders distribution between treatment and placebo group. (Fig 1b). Using the augmented data, we then mined responsive subgroups by searching combinations of features that differentiate treatment effects using recursive partitioning of heterogeneous treatment effects (Fig 1c). We finally identified a complementary set of responsive subgroups for better generalizability and interpretability via rule ensemble (Fig 1d) Our causal clustering method can be used to identify responsive subgroups by the selected rules (Fig 1e).

Therefore, in this paper, we develop an interpretable HTE analysis framework to discover responsive subgroups from randomized data. We propose a novel framework that leverages the ensemble of recursive partitioning to generate initial decision boundaries in terms of treatment effects conditioned on patients’ characteristics and select a set of complementary rules, which helps improve the effectiveness of the treatment plan on the target population. Subjects within a subgroup will share similar characteristics that affect the treatment effects on them, which are interpretable for practice.(Fig 1)

As for the source of randomized data, we focus on completed randomized clinical trials (RCT). Here, a technical challenge is that randomized data usually has a sample size that is too small to support the deep investigation of heterogeneity in subpopulations, which hurts model generalizability and statistical power. [24, 25] Thus, we introduce a data augmentation strategy to help improve the model’s efficacy.(Fig 1b)

## Materials and methods

### Study overview

Based on Neyman-Rubin’s potential outcome framework, we developed an interpretable causal clustering method. Our model was based on the recursive partitioning and rule selection. To overcome the limited sample size to explore heterogeneity, we proposed a data augmentation strategy based on borrowing historical data and generating synthetic data. We applied our method to an ICH clinical trial and demonstrated its ability to derive responsive subgroups with clinical implications. In the following subsections, we will first introduce our causal clustering framework, and then go through the data analysis pipeline for the real-world ICH trial data.

### Notations

- {*X*, *T*, *Y*}: RCT data. *X* = Pre-treatment variables, *T* = Treatment Assignment, *Y* = Outcome;
- {*X*_*B*_, 0, *Y*_*B*_}; $\left\{X_{B}^{\prime },0,Y_{B}^{\prime }\right\}$: Borrowed historical control data; matched historical control data;
-
$\left\{X_{S},T_{S},Y_{S}\right\};\left\{X_{S}^{\prime },T_{S}^{\prime },Y_{S}^{\prime }\right\}$ : Synthetic data; matched synthetic data to real data;
- *τ*(*X*), $\hat{\tau }\left(X\right)$: Heterogeneous treatment effect (HTE); estimated HTE;
- Ω: Split points sets;
- Π, Π_*m*_: Recursive partition on *X*; Recursive partition at depth of *m*;

### Prelinminary: Potential outcome framework

We first revisit preliminaries on the definition of HTE. We follow Neyman-Rubin’s potential outcome framework to define the causal effect of treatment. [26] We make standard assumptions: i) strong ignorability (no hidden confounders), ii) stable unit treatment value (potential outcome of an individual is unrelated to the treatment status of others), and iii) positivity (0 < *P*(*T*|*X*) < 1). Our randomized data is a {*X*, *T*, *Y*} triplet. For each patient, *X* is the feature, *T* is an indicator for treatment assignment and *Y* is an outcome. The factual outcome is the outcome we observe from the data. A counterfactual outcome is a hypothetical outcome under alternative exposure scenarios, thus unobserved. *Y*(*T*) is the outcome when the patient is intervened to being exposed to T. The causal effect of treatment is defined as the difference between factual and counterfactual outcomes. HTE *τ*(*X*) given feature *X* is thus defined as *τ*(*X*) = *E*[*Y*(1) − *Y*(0)|*X*]. However, it is impossible to observe factual and counterfactual outcomes simultaneously. If experiments randomize treatment assignment *T*, an unbiased estimate of *τ*(*X*) can be defined as *τ*(*X*) = *E*[*Y*|*X*, *T* = 1] − *E*[*Y*|*X*, *T* = 0].

### Interpretable HTE estimation

We develop a novel approach that leverages the recursive partitioning for HTE estimation (e.g., causal tree/forest, [27] uplift tree/forest [28, 29]) to generate initial causal decision boundaries and select a set of complementary subgroups via rule selection model. A rule is a conjunction of causal decision boundaries from root to terminal nodes in the tree and is simply a combination of pre-treatment conditions with numerical cutoffs. However, identifying optimal partitioning and, thus, optimal rules requires combinatorial optimization, which is generally infeasible for more than a few variables. We took advantage of an ensemble approach that generates many combinations of rules and selected a complementary set of rules. Patients from a subgroup defined by a set of rules share similar treatment effects, which are interpretable by design and well separated concerning HTE.

#### Responsive subgroup generation

Our objective is to identify “good” recursive partitions of feature space X that the estimated HTE $\hat{\tau }\left(X\right)$ at leaf nodes. We grow an uplift forest to generate candidate rules. [29] The tree algorithm identified splitting criteria that maximize the heterogeneity of $\hat{\tau }\left(X\right)$ by maximizing the difference in outcome distributions between the treatment and the control groups using Kullback-Leibler (KL) divergence. We measure the statistical significance of the rules by the Chi-square test. (Algorithm 1) Details can be found in S1(A) Text.

**Algorithm 1**: Responsive subgroup generation via recursive partitioning

1 GrowTree (*w*)

 **Input** :Root node *w* = {*X*, *Y*, *Z*}

2 Π = []

3 **if** *number of samples in w* < *minimum number of samples OR number of treated samples in w* < *minimum number of treated samples* **then**

4  return Π

5 **else**

6  Among all the features *x*_*i*_, *i* ∈ [1, *n*], find the decision rules Ω(*x*_*i*_) that split the node *w* → *w*_*L*_, *w*_*R*_, such that

$$\underset{x_{i},\Omega \left(x_{i}\right)}{\text{arg}\;\text{max}}\;KL\left(p_{t},p_{c}|\Omega \left(x_{i}\right)\right)-KL\left(p_{t},p_{c}\right)$$

 Π = Π ∪ Ω(*x*_*i*_)

7  GrowTree(*w*_*L*_)

8  GrowTree(*w*_*R*_)

9 **end**

 **Output**: Π

To increase the generalizability and coverage of subgroups, we extract many nodes from an ensemble of uplift trees, which serve as candidates for responsive subgroups. We generated many trees with random bootstrapping to diversify the branches. The HTE is estimated by a weighted average of the estimations from all trees.

#### A complementary selection of subgroups

Although an ensemble of trees may increase the quality of HTE estimation, it may generate redundant or overlapping rules, thus making the subgroups less interpretable. Therefore, after developing an ensemble of trees, we conduct a Chi-square test within each node to check if the outcome distributions in the treatment and control groups are significantly different. We then “flatten” the forest. We extract all significant rules Π_*m*_(*X*) at any depth m from any tree if the Chi-square tests on the nodes give *p* − *value* < 0.05. Our approach to selecting important rules is fitting a *L*1–regularized sparse linear model with the estimated HTE $\hat{\tau }\left(X\right)$ from the ensemble of trees as the outcome, and the rules indicators and original baseline characteristics as the features.(Algorithm 2) Then we can evaluate the effect sizes for the generated rules, as motivated by RuleFit model. [30]

**Algorithm 2**: Complementary selection of subgroups

**Input** :A collection of trees: *F* = {Π_*m*_}_*m*=1,…,*M*_, *α* = 0.05

1 R = []

2 **for** *tree* Π ∈ *F* **do**

3  **for** *node m* ∈ Π **do**

4   *p* − *value* ← *χ*^2^ − *test* on Π_*m*_(*X*)

5   **if** *p* − *value* < *α* **then**

6    *R* ← *R* ∪ [Π_*m*_]

7   **else**

8    Continue

9   **end**

10  **end**

11 **end**

 /* Maps the original features *X* to the rules features Π.    */

12 Π = Π_*m*_(*X*)

 /* Extract the estimation from the Forest *F* as the outcome for the sparse linear model     */

13 $\hat{\tau }\left(X\right)=F\left(X\right)$

14 return *a*_*m*_ and *b*_*d*_ such that

$$\text{arg}\;\underset{a_{m},b_{d}}{\text{min}}\;|\hat{\tau }\left(X\right)-\left[\sum_{\Pi_{m}\left(X\right)\in R}a_{m}\Pi_{m}\left(X\right)+\sum_{X_{d}\in X}b_{d}X_{d}+c\right]{|}^{2}+\lambda (\sum (|a_{m}\left|+\right|b_{d}\left|)\right)$$

### Data augmentation

A major obstacle to deploying this model is that most RCT data have a small sample size, which limits the extent of exploring heterogeneity within the population. The small sample size of RCTs is mainly due to cost constraints, such as the time and effort required for participant recruitment and retention, and ethical concerns. To address the challenge, we leveraged two strategies: (i) borrowing historical controls from external observational data and (ii) generating similar but synthetic data.

The first strategy is to use data from patients who received standard care in previous studies as a control group to increase the sample size of RCT. [31, 32] The critical assumption underlying this technique is that patients in the historical control group are comparable to those in the RCT concerning important clinical variables that may influence the primary outcome. To ensure this, we carefully selected historical controls {*X*_*B*_, 0, *Y*_*B*_} following the same eligibility criteria of the RCT population.

As the first strategy can only increase the sample size of the control group, we implement another strategy that helps augment both arms. The idea is to train a generative model to learn the real data’s distributions and draw high-quality samples that are hard to distinguish from the real data. Generating synthetic tabular data has been widely studied. [33–36] In our study, we tried the conditional tabular data generative adversarial network (CTGAN) and Tabular Variational Autoencoders (TVAE) (S1(B) Text). [36] We trained the generative model using all real data {*X*, *T*, *Y*} and {*X*_*B*_, 0, *Y*_*B*_}, as larger training data lead to higher performance of the generative model and can also increase the heterogeneity of synthetic samples. We evaluated synthetic data quality by the Kolmogorov-Smirnov test and the total variation distance(TVD).

Our framework was built on an uplift forest, which works under the assumption that the data is randomized, while the data augmentation strategy introduces confounding biases to the training data. We introduced a propensity score matching (PSM) strategy to address the confounding biases. In detail, matched the augmented data *X*_*B*_, 0, *Y*_*B*_ or *X*_*s*_, *T*_*s*_, *Y*_*s*_ to the real RCT data *X*, *T*, *Y* using propensity scores to ensure the balance of pre-treatment variables. Specifically, to match the borrowed historical controls {*X*_*B*_, 0, *Y*_*B*_} to the real RCT data *X*, *T*, *Y*, we trained an Elastic Net with regularization on all the data {*X*, *T*, *Y*} + {*X*_*B*_, 0, *Y*_*B*_} to estimate propensity scores, and then performed a 1:1 nearest neighbor matching between the RCT’s treatment arm *X*, 1, *Y* and the borrowed control arm {*X*_*B*_, 0, *Y*_*B*_} to get similar subjects. We denote the matched borrowed data as $X_{B}^{\prime },0,Y_{B}^{\prime }\}$. To match the synthetic data to real data, we applied the nearest neighbor matching by developing propensity score matching models to match the real treated subjects {*X*, 1, *Y*} with the synthetic control subjects {*X*_*S*_, 0, *Y*_*S*_}, and to match the control subjects of the real data $\left\{X,0,Y\right\}+\left\{X_{B}^{\prime },0,X_{B}^{\prime }\right\}$ with the synthetic treated subjects *X*_*S*_, 1, *Y*_*S*_. We denote the matched synthetic data as $\left\{X_{S},T_{S}^{\prime },Y_{S}^{\prime }\right\}$.

### Application to the ATACH2 trial

ATACH2 is a randomized clinical trial to evaluate the treatment effect of the medical intervention of intensive blood pressure (BP) lowering therapy. [6] Participants included in this trial are first-time ICH patients who had systolic blood pressure > 180 mm hg at admission and hematoma volume < 60 ml. The primary outcome is the modified Rankin scale (mRS) score measured around 90 days after randomization. ERICH is an observational clinical trial to observe ICH patients. [37] The participants receive the standard-of-care intervention. ERICH contains all types of spontaneous ICH patients. To include only comparable patients, we selected ERICH patients who meet ATACH2’s eligibility criteria: no prior ICH and the ICH confirmed at first CT after onset, which gives us 2,706 ICH patients out of 3,000. Baseline characteristics are shown in Table 1.

**Table 1:** Baseline characteristics of the real-world trials’ data.

	ATACH2	ERICH
Treatment arm, #	500	0
Control arm, #	500	2,706
**Demographics**		
Age	62 (52, 71)	60 (51, 73)
**Race/Ethnicity, #**		
White	287 (28.7%)	877 (32.4%)
Black	133 (13.3%)	907 (33.5%)
Hispanic	79 (7.9%)	922 (34.1%)
Asian	562 (56.2%)	NA
**Gender, #**		
Male	620 (62.0%)	1,591 (58.8%)
Female	380 (38.0%)	1,115 (41.2%)
**Condition**		
**ICH Location, #**		
Lobar	108 (10.8%)	792 (29.3%)
Basal Ganglia	507 (50.7%)	739 (27.3%)
Thalamus	373 (37.3%)	741 (27.4%)
ICH volume, ml	11.2 (6.1, 19.5)	11.3 (4.2, 27.5)
IVH volume, ml	0.0 (0.0, 0.7)	0.0 (0.0, 0.5)
GCS score	15.0 (13.0, 15.0)	15.0 (13.0, 15.0)
NIHSS score	11.0 (7.0, 16.0)	NA
SBP, mmHg	174.0 (159.0, 190.0)	186.0 (159.0, 215.0)
DBP, mmHg	93.0 (81.0, 106.0)	101.0 (85.0, 120.0)
**Lab test results**		
WBC	7.0 (6.0, 9.0)	8.0 (6.0, 11.0)
Hemoglobin	14.0 (13.0, 15.0)	13.0 (12.0, 15.0)
Hematocrit	42.0 (39.0, 45.0)	NA
PC	214.0 (180.0, 256.0)	221.0 (177.0, 268.0)
APTT	27.8 (24.8, 31.0)	28.2 (25.8, 31.5)
INR	1.0 (0.9, 1.0)	1.0 (0.9, 1.1)
Glucose	122.0 (106.0, 150.0)	132.0 (108.0, 168.0)
Sodium	140.0 (138.0, 142.0)	NA
Potassium	3.8 (3.5, 4.1)	NA
Chloride	104.0 (102.0, 106.0)	NA
CD	25.7 (23.5, 27.3)	NA
Blood Urea Nitrogen	15.0 (12.6, 19.0)	NA
Creatine	0.9 (0.7, 1.1)	NA
**Medical Histories**		
Hypertension	793 (79.3%)	2,163 (79.9%)
Hyperlipidemia	241 (24.1%)	804 (29.7%)
Type I Diabetes	11 (1.1%)	32 (1.2%)
Type II Diabetes	175 (17.5%)	583 (21.5%)
Heart Failure	37 (3.7%)	194 (7.2%)
Atrial Fibrillation	36 (3.6%)	250 (9.2%)
Peripheral Vascular Disease	22 (2.2%)	65 (2.4%)
Cocaine	NA	65 (2.4%)
Smoking	NA	1,075 (39.7%)
PTCA	44	103 (3.8%)
Myocardial infraction	1 (0.1%)	143 (5.3%)
**Outcome**		
mRS score ≥90 days	3.0 (1.0, 4.0)	3.0 (1.0, 4.0)

Mean(overall percentage) and interquartile range(e.g., 50%(25%, 75%)). ICH = intracerebral hemorrhage, IVH = intraventricular hemorrhage, GCS = Glasgow Coma scale, NIHSS = NIH Stroke Scale, SBP = systolic blood pressure, WBC = White Blood Count, PC = Plate Count, APTT = Activated Partial Thromboplastin Clotting, International normalized ratio (INR)

We harmonized the two trials by resolving different granularity of brain locations and units. We log-transformed features with skewed distribution and did normalization for variables with large variance. We used miceforest [38] to impute 3,706 subjects with 3 iterations, gradient boosting decision tree method with at least 20 samples in leaves.

**Fig 2:**
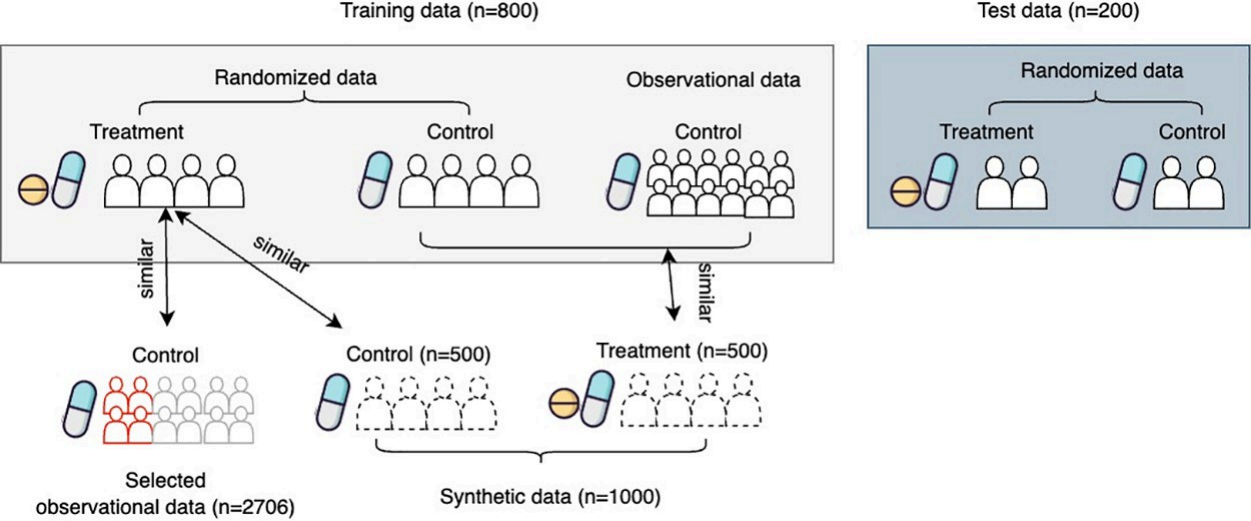
Workflow of the data augmentation strategy.

We tried two tabular data models for synthetic data augmentation: TVAE and CTGAN. We trained the TVAE and CTGAN with the default parameters (300 epoch and batch size of 500, the dimensions of embedding layers, compression layers, and the decompression layers are all 128) on the training dataset and generated synthetic data with 500 treatments and 500 controls. We performed 1:1 PSM with a caliper of 0.2 standard deviations of the variables. Unmatched patients from real data were kept in the cohort after matching. We evaluate data balance after matching by Standard Mean Difference (SMD). The workflow of augmenting the data is shown in Fig 2.

Following the original study statistical analysis setting, the primary endpoint was the mRS measured around 9 months after randomization and binarized as 1 if mRS score≤ 2 and 0 otherwise. [6] A higher mRS means severe disability, so the responsive subgroups should have HTE> 0. All the datasets for augmentation, including ERICH $\left\{X_{B}^{\prime },0,Y_{B}^{\prime }\right\}$ and synthetic data $\left\{X_{S}^{\prime },T_{S}^{\prime },Y_{S}^{\prime }\right\}$ sets, were only used in training. The maximum depth of the tree is fixed at 3 as we only want to keep interaction terms of at most 2 features for interpretation. Each experiment was repeated 30 times with different random seeds to train the model. The hyperparameters of the models are determined by a 4-fold cross-validation. We refer to the Qini-coefficients to evaluate and do model selection; details are introduced in S1(C) Text.

## Results

### Data pooling summary

We reported the number of treated and control samples in each cohort. 200 samples were randomly drawn from ATACH2 as the test data. To address the potential confounding bias by pooling the data from two studies, we performed a 1:1 PSM. We reported the cohort size, SMD, and the AUC for distinguishing between treated and control patients before and after matching in Table 2. The average SMD between the confounders of the treatment and control arms was 0.0605 after matching, and the AUC to distinguish the treatment and control group decreased from 0.9183 to 0.6539 (Table 2), showing adequate balance between the treatment and control groups. After data augmentation, the training dataset contains 1741 subjects (800 from ATACH2, 134 from ERICH, and 807 from synthetic data).

**Table 2:** Balance in confounders from different training data. AUCs before and after matching were reported.

	Metric (Direction of favorable score)						
	Data size (↑)			SMD between two arms (↓)		AUC to distinguish between two arms (↓)	
	Treatment	Control	Total	Before matching	After matching	Before matching	After matching
Randomized data (Training)	397	403	800	0.0485	-	0.6208	-
Randomized data (Training)+Histroical control	397	537	934	0.2661	0.0605	0.9183	0.6539
Randomized data (Training)+Historical control+Synthetic data(TVAE)	492	682	1,174	0.2597	0.0868	0.8419	0.6901
Randomized data (Training)+Historical control+Synthetic data(CTGAN)	733	856	1,589	0.0416	0.0216	0.5890	0.5426

Lower AUC values are favorable as confounders should not distinguish between treatment or placebo groups.

**Fig 3:**
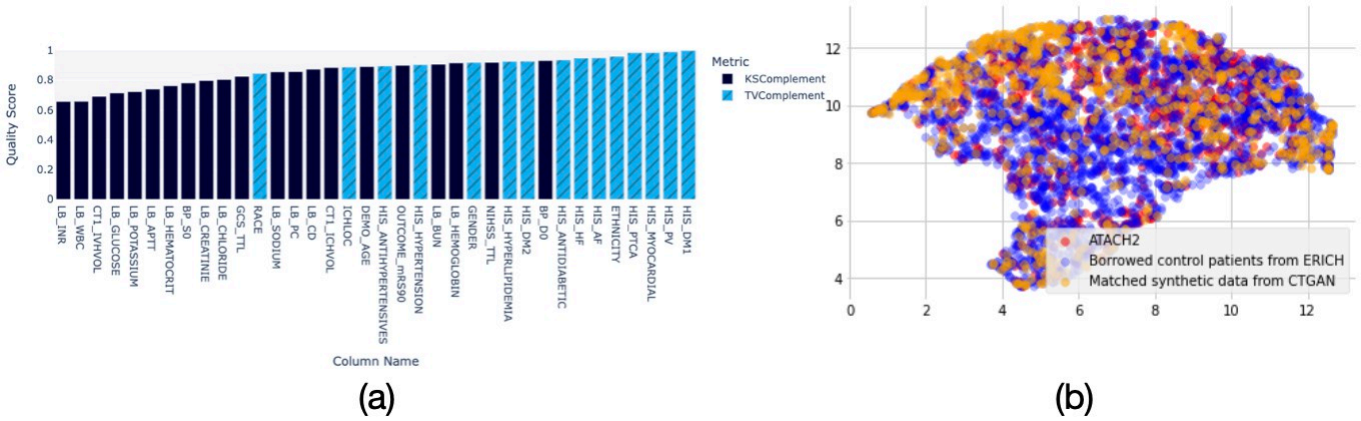
The similarity between real and synthetic data. (a). Individual variable’s distribution similarity. Dark blue: continuous variables, evaluated by KS statistics; Light blue: Categorical variables, evaluated by TVD. (b). UMAP plot of individual samples from ATACH2 borrowed historical control from ERICH, matched synthetic samples generated by CTGAN.

We created 1000 synthetic subjects, 500 in the treatment and 500 in the control groups. The sample size of the synthetic dataset was determined by grid search with fixed hyperparameters. We compared the synthetic data to the real data from ATACH2 and ERICH trials. We found that the individual variable’s similarity score was above 0.7 for all variables except INR, WBC values, and IVH volume (Fig 3a). This suggests that the synthetic data’s distribution is close to the target data.

We performed PSM on the synthetic data to maintain the balance of the baseline characteristics while increasing the same size in an unbiased manner, which resulted in 386 synthetic control and 421 synthetic treatment data. The matched synthetic data resulted in a decreasing SMD from 0.0416 to 0.0216 and a decrease in the AUC for discriminating arms from 0.5890 to 0.5426 (Table 2). The SMDs of all the baseline features after matching were lower than 0.1 after PSM, which is considered balanced between the treatment and the control groups. Also, the UMAP shows that the matched synthetic and real data were indistinguishable when comparing the distribution of individual samples on a high dimensional space (Fig 3b). In comparison, using the TVAE model, another synthetic data generation model, we got a matched cohort of 1174 subjects that has an average SMD of 0.0868, and the AUC for discriminating arms is 0.6901 (Table 2). This suggests that the CTGAN model can help augment data with similar data as the target trial after PSM.

### Model’s utility and interpretability

Table 3 shows the evaluation of the model’s estimation quality and interpretability. We evaluated the estimation quality by Qini-coefficient and evaluated interpretability by the number of significant rules (i.e., the total number of important rules generated and selected by our model given different strategies). A desirable model would have high estimation quality and could also pick out the most significant rules using a data-driven method.

**Table 3:** HTE estimation quality and interpretability with augmented data (Randomized data + historical control + CTGAN synthetic data). Mean Qini-coefficient and standard deviation by repeating experiments 30 times with different random seeds.

	Uplift Forest	Uplift Forest+Rule selection model
Qini-coefficient(↑)	0.1823 ± 0.0296	0.1822 ± 0.0296
The number of rules with p-value < 0.05 generated from uplift forest v.s. number of rules selected by different strategies from rule selection model(↓)	221.3 ± 6.5	*a*_*m*_ ≠ 0: 195.4 ± 5.5
		|*a*_*m*_|> 0.005: 9.7 ± 2.4
		Significance Score> 0.002: 3.6 ± 1.3

Regarding estimation quality, the uplift forest and the rule selection model achieved the Qini-coefficient of 0.1823 and 0.1822, respectively, implying that adding a regularized linear model does not affect the model’s performance in ranking the patients by treatment effect size. In assessing interpretability, we illustrated the distributions of coefficients, support, and importance scores for rules generated by models with varying random seeds, as depicted in S2 Fig. These histograms indicate that the number of chosen rules declines upon reaching specific thresholds. For instance, S2(A) Fig reveals a noticeable reduction in the number of rules with absolute coefficient values exceeding 0.005 and a significant decline in rules with values surpassing 0.002. On average, there are 195.4 rules with coefficients≠ 0, 9.7 with coefficients greater than 0.005, and 3.6 with significance scores above 0.002. Utilizing a sparse linear model can modestly decrease the rule count, but ruleset refinement is further achieved by employing various rule selection strategies.

**Fig 4:**
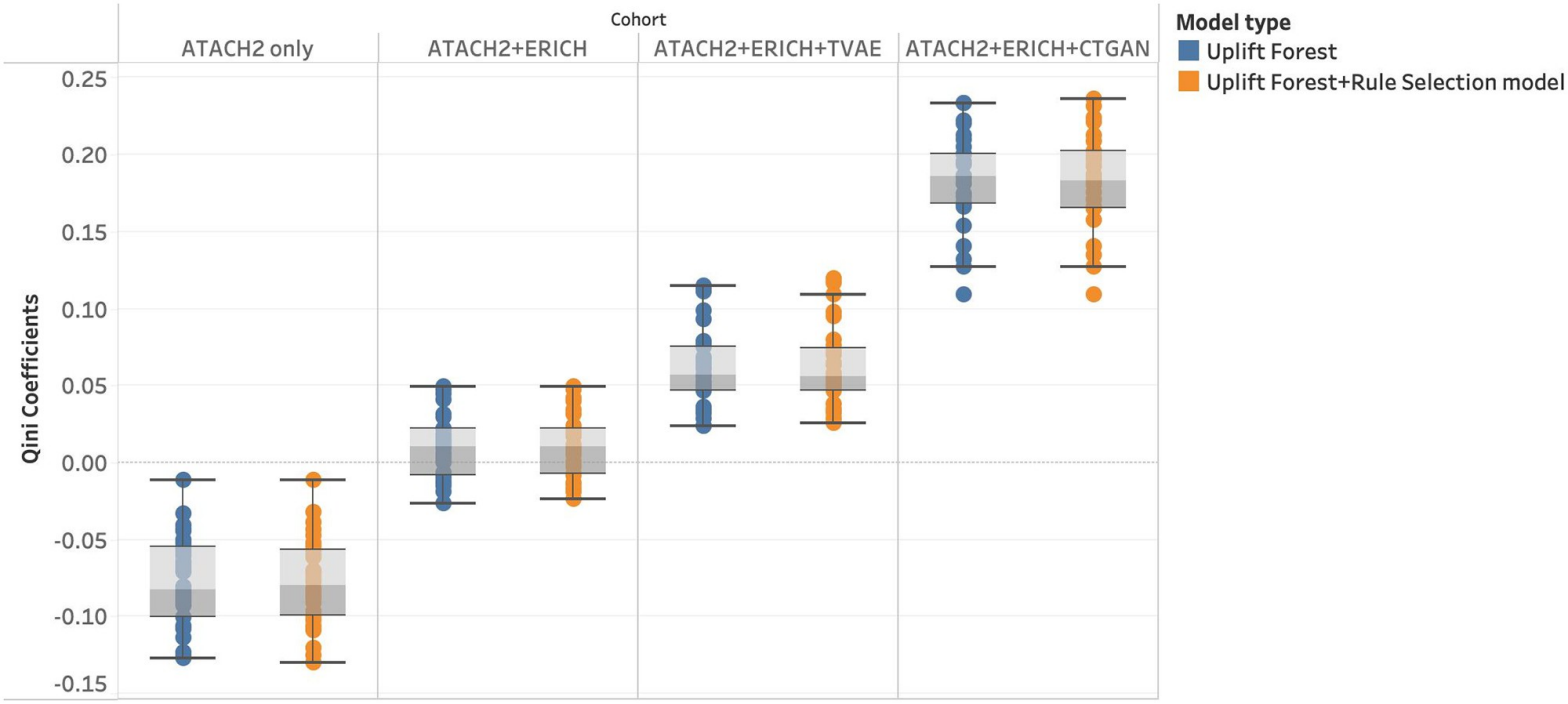
HTE estimation quality by different data augmentation methods. R = Randomized data only, R+HC = Randomized data+historical control, R+HC+TVAE = Randomized data + historical control + synthetic data (TVAE), R+HC+CTGAN = Randomized data + historical control + synthetic data (CTGAN).

Fig 4 compares models’ estimation quality trained on different cohorts. The results show that the model trained on the cohort augmented by historical control and synthetic data from the CTGAN model achieves the highest Qini-coefficient, 0.1822 ± 0.0256, while with the synthetic data from TVAE model, the model can achieve the Qini-coefficients of 0.0614 ± 0.0252.(Fig 4)

### Finding: A complementary set of salvageable subgroups in ATACH2

We picked the best model trained on the cohort of ATACH2, ERICH, and synthetic data from the CTGAN model. The best model achieved the Qini-coefficient 0.2363. The estimated HTE of the test datasets ranges from -0.1225 to 0.0868 (Mean = 0.0350, IQR = -0.021, -0.002, 0.012).

**Fig 5:**
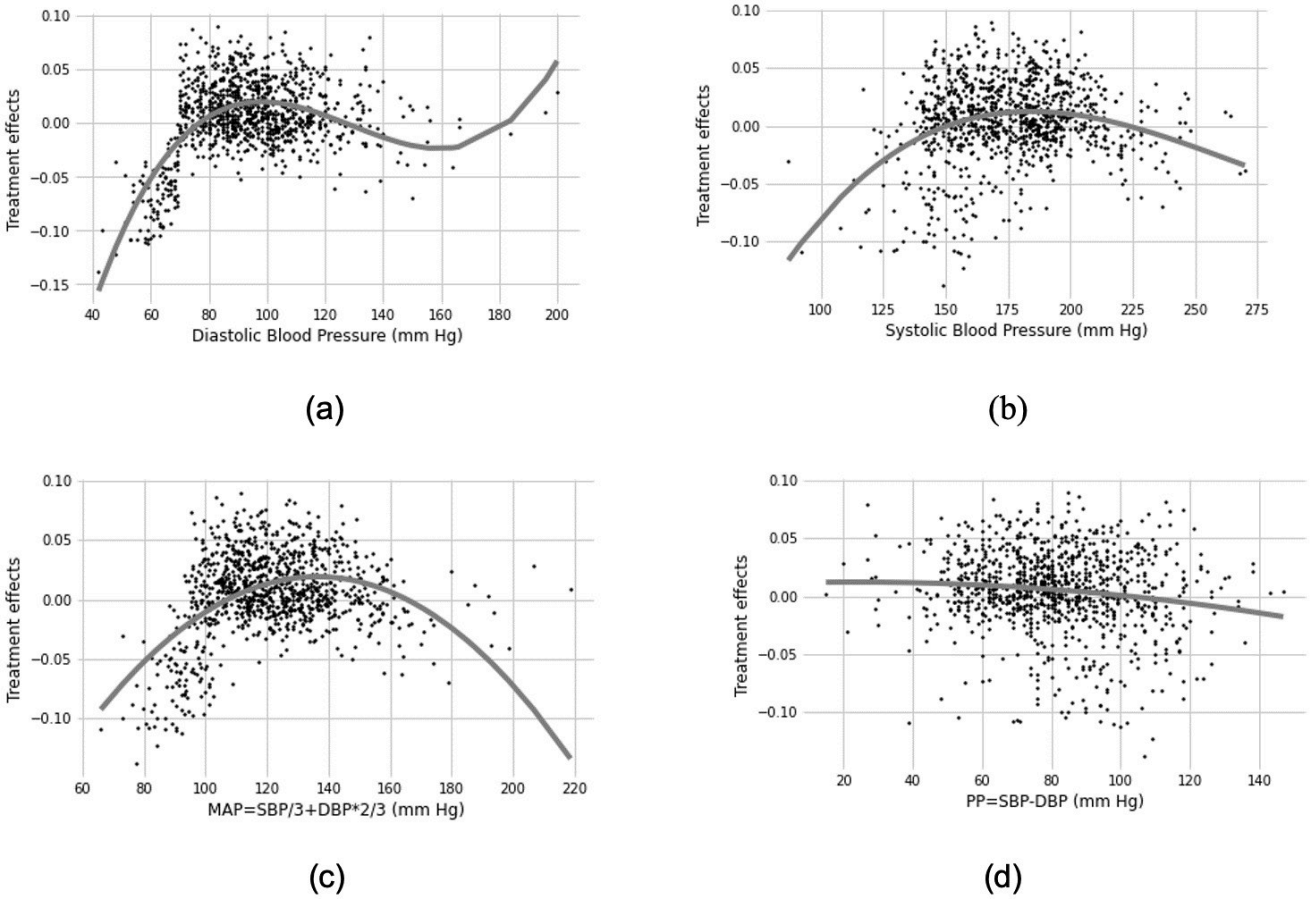
Relationship between the intensive blood pressure therapy’s efficacy and blood pressure measurements. (a). Systolic blood pressure(SBP); (b). Diastolic blood pressure(DBP); (c). MAP: *MAP* = *SBP*/3 + *DBP* × 2/3; (d) PP: *PP* = *SBP* − *DBP*. The gray shows the polynomial regression fitted curve between the selected covariates and the estimated treatment’s efficacy.

Using this model, we ranked all the covariates, including original features and combinations, according to their importance scores. Table 4 shows the top 5 subgroups in which patients benefit more from the intensive blood pressure therapy plan and the top 5 subgroups in which the patients benefit more from the standard blood pressure reduction therapy. The estimated coefficients of the features and their combination indicate how much it will affect the treatment effect size. Also, in the context of clinical experience, blood pressure-related measurements are directly linked to the treatment and the outcome. Thus, we investigated the relationship between the blood pressure-related measurement and the predicted treatment effects from our model, and we fit a polynomial regression model to show the trend for each of them (Fig 5). In Fig 5a and 5c, we can observe an obvious increment of treatment effects with DBP at around 80 mm hg and SBP at around 100 mm hg. Also, Fig 5b illustrates that patients with baseline SBP within a certain range (e.g. 150–200 mm hg) tend to benefit more from intensive blood pressure therapy.

**Table 4:** Top 5 important rules that increase the treatment effects and top 5 rules/features that decrease the treatment effects of intensive blood pressure reduction therapy (ranked by importance score).

Subgroup description	Coefficient	Support, %	Importance	ATE within subgroup (On test data)
Favorable treatment effects				
DBP≥ 70 and SBP< 215	0.0090	1,426 (81.91%)	0.0035	0.0138 ± 0.0238
DBP≥ 70 and ICH located in Basal Ganglia or Lobar or Thalamus	0.0084	1,479 (84.95%)	0.0030	0.0137 ± 0.0234
NIHSS≥ 3.0 and DBP≥ 70.0	0.0057	1,444 (82.94%)	0.0022	0.0144 ± 0.0231
Homoglobin< 12.5 and DBP≥ 70.0	0.0054	296 (17.00%)	0.0020	0.0298 ± 0.0255
DBP≥ 70.0 and DBP< 147.0	0.0052	1,489 (85.53%)	0.0018	0.0134 ± 0.0255
Unfavorable treatment effects				
Homoglobin≥12.5 and ICH location not in Basal Ganglia	-0.0046	896 (49.91%)	0.0023	−0.0039 ± 0.0365
DBP< 70	-0.0063	159 (9.13%)	0.0018	−0.0644 ± 0.0315
DBP< 64	-0.0077	83 (4.77%)	0.0016	−0.0822 ± 0.0303
MAP< 104.0 and CD≥ 23.2	-0.0041	244 (14.01%)	0.0014	−0.0261 ± 0.0480
PC≥ 191.0 and Potassium≥ 3.2	-0.0028	1,106 (63.53%)	0.0013	−0.0055 ± 0.0308

NNT represents the total number of data points that fall in this subgroup in the training set; Subgroup ATE is estimated on all the ATACH2 data.

## Discussion

In this study, we proposed a framework for automatically identifying responsive subgroups from real-world RCT data. We generated candidate rules using an ensemble of recursive partition algorithms and employed a regularized linear model for complementary rule selection. Given the limited sample size of the RCT, we embraced a data augmentation strategy that tapped into both external observational study data and synthetic data. The proposed approach amplifies our model’s efficacy in analyzing the RCT data and augments the statistical power. Additionally, we considered the potential confounding bias introduced by the external data by employing a matching strategy during the data augmentation process. We applied our model to an ICH clinical trial and demonstrated its ability to derive responsive subgroups with clinical implications.

### Methodological findings

#### Interpretable clustering by rule selection

Our approach is inspired by the RuleFit algorithm. [30] Initially, RuleFit was designed for traditional regression and classification tasks. We adapted it for HTE estimation. This method allows us to pinpoint crucial combinations of moderators stratified by a threshold, leading to the identification of interpretable subgroups with similar treatment effects. From the results, we learn that the LASSO model does not help improve the performance of uplift modeling which differs in characteristics from RuleFit designed for classic regression or classification tasks. The possible reason is that we train the sparse linear model on a sudo-label in the second step as the true label is unavailable in the treatment effects estimation task. This idea is similar to meta-learner. [20] Further work could explore boosting the performance of meta-models for uplift modeling tasks.

#### Data augmentation

The data augmentation approach we employed was motivated by the limited sample size of clinical trial datasets, making it challenging to capture heterogeneity among the population. In this paper, we first augment the dataset with real data from other studies. Then, we introduced a synthetic augmentation procedure to increase the sample size of the training set. This study delved into two of the most state-of-the-art tabular generation models: CTGAN and TVAE (S1(B) Text). Our findings indicate that CTGAN outperforms TVAE in mimicking real-world data, especially in representing rare categories in highly imbalanced categorical variables (S1 Fig). As to the downstream task of estimating HTE, we can learn from the results that the model trained on the data augmented by CTGAN performs better than that augmented by TVAE. This disparity might stem from CTGAN’s learning multiple modes in continuous variables and the highly imbalanced categorical variables of the tabular data.

Interestingly, our post-hoc analysis revealed that amplifying synthetic data volume doesn’t necessarily boost our model’s efficacy (S3 Fig). It is because we introduce a matching procedure to balance the cohort, inherently restricting the matched cohort size due to the finite sample size of the real-world data. Also, as discussed in another study that utilized the synthetic data augmentation procedures, the phenomenon is possibly caused by the model’s mode collapse issue. [39] Currently, no study discussed the synthetic method for downstream tasks of causal effects estimation, leaving us with an unanswered question of which characteristics of the synthetic data will affect the evaluation metrics for causal models. Further exploration is necessary to fully understand the nuances of synthetic data augmentation in the context of RCTs and answer causal questions.

### Clinical implication of findings

The ATACH2 trial was not able to demonstrate a decrease in disability and mortality in the treatment group. Our findings suggest that there are subgroups that could benefit from aggressive blood pressure lowering in whom this intervention may be safe and effective. We also identified subgroups that may have worse outcomes with a targeted systolic blood pressure of 110–139 mm hg.

Table 4 shows that the subgroups that benefited most from intensive blood pressure lowering include patients with DBP≥ 70 mm hg and SBP< 215 mm hg. This shows that there may be an optimal blood pressure range where patients may benefit from intensively lowering blood pressure. This includes patients whose SBPs are not extremely high (SBP< 215 mm hg) as such large drops in blood pressure may contribute to worsened outcomes. This is in line with the post hoc analysis of the ATACH2 trial which used a cut off 220 mm hg and showed that intensive control of BP in patients with SBP higher than 220 mm hg led to poorer outcomes. [40] While the literature on intensive blood pressure control in patients with DBP is limited, DBP contributes to cerebral perfusion pressure, and further aggressive lowering beyond diastolic DBP < 70 mm hg may lead to decreased brain perfusion and hence worse outcomes.

High PP has been independently linked to worse outcomes.(Fig 5) This has been hypothesized to be secondary to the disruption of autoregulation leading to increased dependence on higher MAPs to ensure cerebral perfusion. Thus, if the blood pressure is actively lowered as part of the treatment, these patients will do worse.

Anemia has been independently linked to poorer outcomes after ICH. However, the interaction between hemoglobin levels on admission and blood pressure lowering remains unclear. Fig 5 suggests that the lowest negative treatment was at systolic blood pressures between 150–200 mm hg, diastolic BP of 70–140 mm hg, MAPs in the 100–150 mm hg range, while increasing PP may lead to worse outcomes as suggested earlier. These presenting blood pressure ranges offer reasonable drops in blood pressure without causing large changes in PP and hence may be where aggressive BP lowering is most effective. Similar rules were identified by comparing mRS score≥3 and mRS score< 3 (S1 Table).

### Limitations

However, the study’s findings must be interpreted within several limitations. First, our framework was based on the assumption that the data is randomized. Using a data augmentation strategy, the randomization feature of the trial data is no longer kept. Although we performed PSM to simulate the randomization, there might be unobserved confounders in the augmented data. Moreover, the uplift forest is a basic model in the uplift modeling field which is easy to implement and interpret, while it has a limited ability in terms of quality of estimated HTE. Future work could explore advanced algorithms for generating decision rules to improve the model’s performance while maintaining utility and interpretability. It is also important to note that while our methodology identifies important subsets, the effect size is small, and clinical relevance needs further studies.

## Conclusion

The proposed framework helps identify several responsive subgroups regarding HTE in a comprehensive decision rule format. By doing data augmentation with data from different resources, we improved the model’s performance in terms of Qini-coefficient compared with the model trained on the trial data only. The model of the best evaluation metric gives rules of good quality from a clinical perspective and coincides with many other studies’ findings of the therapy plan for intracerebral hemorrhage. This work provides a foundation for mining information regarding causal effects from failed trials which helps develop new trials and treatment plans.

## Supporting information

S1 Text Detailed descriptions of methods. (A). Details about subgroup generation algorithm (B). Introduction to CTGAN and TVAE (C). Details of the propensity score matching in the ATACH2 study (D). Introduction to Qini-coefficient and Importance Scores.
(DOCX)

S1 Fig Diagnosis for the synthetic data from TVAE model and the CTGAN model. (PNG)

S2 Fig Distributions of effect sizes and importance scores of different random seeds. (PNG)

S3 Fig Synthetic sample size’s effect on: (A) model’s performance; (B) the number of matched synthetic data points. (PNG)

S1 Table Top rules for comparing mRS score ≥ 3 v.s. mRS score < 3. The Qini-coefficient of the model is 0.1271.
(DOCX)
