# Supplementary material for: An interpretable framework to identify responsive subgroups from clinical trials regarding treatment effects: Application to treatment of intracerebral hemorrhage
Source: PLOS Digit Health. 2024 May 7;3(5):e0000493. doi: 10.1371/journal.pdig.0000493 (PMC11075857; doi:10.1371/journal.pdig.0000493)
Supplement: S1 Text — (A). Details about subgroup generation algorithm (B). Introduction to CTGAN and TVAE (C). Details of the propensity score matching in the ATACH2 study (D). Introduction to Qini-coefficient and Importance Scores. (DOCX) [file pdig.0000493.s001.docx]

**Supplementary Material A. Details about subgroup generation algorithm**

Our objective is to identify a “good” recursive partition of feature space $X$ that the estimated HTE $\hat{\tau}(X)$ at leaf nodes is generalizable to unseen data. We recursively split nodes by selecting one feature and splitting by $\Omega$ until the number of patients in the node reaches the minimum level. Identifying the best node-splitting criteria $\Omega$ for the treatment effect is different from identifying node-splitting in the typical decision tree for classification or regression, as the ground-truth treatment effect $\tau(X)$ is never observed. Instead, causal tree or uplift tree maximizes the heterogeneity of  $\hat{\tau}(X)$ across nodes.[1], [2] We grow an uplift forest to generate candidate rules.[3] We identified splitting criteria that maximize the heterogeneity of $\hat{\tau}(X)$ by maximizing the difference in outcomes distribution between the treatment group and the placebo group. We used Kullback-Leibler (KL) distance to measure the differences:

$$KL(p_{t},p_{c})=\sum_{y} p_{t}\left( y \right)log\frac{p_{t}(y)}{p_{c}(y)}$$

where $p_{t}$ and $p_{c}$ is the distribution of the outcome $Y$ of the treatment and placebo groups, respectively. Given a splitting strategy $\Omega$, the parent node $w$ is split into left $w_{L}$ and right $w_{R}$ child nodes. The distance is then updated as:

$$KL(p_{t},p_{c}|\Omega)=\frac{N\left( w_{L} \right)}{N}KL(p_{t}(y|w_{L}),p_{c}(y|w_{L}))+\frac{N\left( w_{R} \right)}{N}KL(p_{t}(y|w_{R}),p_{c}(y|w_{R}))$$

where $N$, $N(w_{L})$ and $N(w_{R})$ are the total number of subjects of the current node, left, and right child nodes respectively. We aim to find the split $\Omega$ at each node that maximizes the treatment effect increment after splitting:

${argmax}_{\Omega}KL(p_{t},p_{c}|\Omega)-KL(p_{t},p_{c})$.

Then the final partition $\Pi$ has the form of a conjunctive feature stratification that involves all splitting criteria $\Omega_{j}$ along the nodes *j* from root to a terminal node m:

$$m(X)=j\{root,..m\} I(j),$$

where $I(\cdot)$ is an indicator that denotes 1 if x satisfies $\Omega$ and 0 otherwise. $\Pi_{m}(X)$ is 1 if the features $X$ satisfy all the conditions $\Omega$. We used the partition $\Pi$ to define subgroups.

**Supplementary Material B. Introduction to CTGAN and TVAE**

CTGAN is a Generative Adversarial Network that used for synthesizing tabular data.[4] It includes a conditional generator and is trained by sampling the all the categories from the discrete columns to learn the conditional distribution of other variables conditioned on one categorical attribute. This allows the model to better capture the correlations between different categories in categorical features and can also recover the true distribution of real data and the highly imbalance characteristics of some discrete columns. In addition, CTGAN uses a specific loss function, called the Wasserstein loss with gradient penalty, which helps the model to train more effectively.

TVAE is another sophisticated generative model that is designed for tabular data synthesis.[4] It's a Variational Autoencoder (VAE). It is composed of an encoder and a decoder which tries to model the latent distribution, $p(z_{j}|r_{j})$,and latent representation, $p(r_{j}|z_{j})$, respectively. The model is trained by minimizing the evidence lower bound (ELBO) loss.

Both GAN and VAE are widely applied to synthetic data generation tasks. For the two models we used in our study, there are some differences that makes them perform differently on our data. The TVAE used in our work is a classical VAE architecture which tries to learn a latent space for reconstructing samples. But as pointed out by the original study[4], it is hard for the classic model to deal with multiple modes in continuous variables and the imbalance issue in the discrete variables. Instead, the CTGAN model accommodate a conditional generator to deal with the highly imbalanced categorical variables and uses the mode normalization techniques to deal with the continuous variables with complex distributions. We observed the characteristics of the two synthetic data generation models.

**Supplementary Material C. Introduction to Qini-coefficients and Importance scores**

We evaluated the HTE estimation quality of causal tree, forest, and the causal clustering model using Qini-coefficient.[3], [5] The Qini-coefficient measures the HTE estimation model’s ability to sort the samples based on their treatment effect size. The Qini-coefficient evaluates cumulative benefits as we increase the fraction of samples that are sorted by estimated HTE in descending order. That is,

$$Qini(q)=Y_{T}(q)-\frac{Y_{C}\left( q \right)N_{T}\left( q \right)}{N_{C}\left( q \right)},$$

where $Y_{T} (q)$ represent the sum of outcomes in samples in the treatment $T$ group in the *q* quantile, and $N_{T}(q)$ is the total number of samples in the treatment $T$ group in the *q* quantile (the same for placebo $C$ group). Thus $Qini(q)$ measure the difference in outcomes between treatment and placebo groups for samples in the first *q* quantile. The $Qini(q)$ would be large at the earliest quantile (as the estimated HTE is large) and decrease gradually as increasing the quantiles (as the estimated HTE is small). The Qini-coefficient is the area under the $Qini(q)$ curve, and the value is large if a model can rank the samples with favorable outcome ahead of the unfavorable outcome by estimated HTE.

**Importance score of responsive subgroups.** After we evaluated the quality of the proposed HTE estimation model, we selected important rules (or subgroups) using coefficient am of LASSO model and support s of the rule $\Pi_{m}(X)$. We calculate a importance score of a rule $\Pi_{m}(X)$ by $\left| a_{m} \right|\cdot\sqrt{s(1-s)}$.[6]

# Reference

[1] S. Athey and G. Imbens, “Recursive partitioning for heterogeneous causal effects,” *Proc. Natl. Acad. Sci.*, vol. 113, no. 27, pp. 7353–7360, Jul. 2016, doi: 10.1073/pnas.1510489113.

[2] N. J. Radcliffe and P. D. Surry, “Real-World Uplift Modelling with Signiﬁcance-Based Uplift Trees,” 2011.

[3] L. Guelman, M. Guillén, and A. M. Pérez-Marín, “Uplift Random Forests,” *Cybern. Syst.*, vol. 46, no. 3–4, pp. 230–248, May 2015, doi: 10.1080/01969722.2015.1012892.

[4] L. Xu, M. Skoularidou, A. Cuesta-Infante, and K. Veeramachaneni, “Modeling Tabular data using Conditional GAN”.

[5] M. Belbahri, A. Murua, O. Gandouet, and V. P. Nia, “Qini-based uplift regression,” *Ann. Appl. Stat.*, vol. 15, no. 3, pp. 1247–1272, Sep. 2021, doi: 10.1214/21-AOAS1465.

[6] J. H. Friedman and B. E. Popescu, “Predictive learning via rule ensembles,” *Ann. Appl. Stat.*, vol. 2, no. 3, pp. 916–954, Sep. 2008, doi: 10.1214/07-AOAS148.
