# Supplementary figures and images for: An interpretable framework to identify responsive subgroups from clinical trials regarding treatment effects: Application to treatment of intracerebral hemorrhage
Source: PLOS Digit Health. 2024 May 7;3(5):e0000493. doi: 10.1371/journal.pdig.0000493 (PMC11075857; doi:10.1371/journal.pdig.0000493)

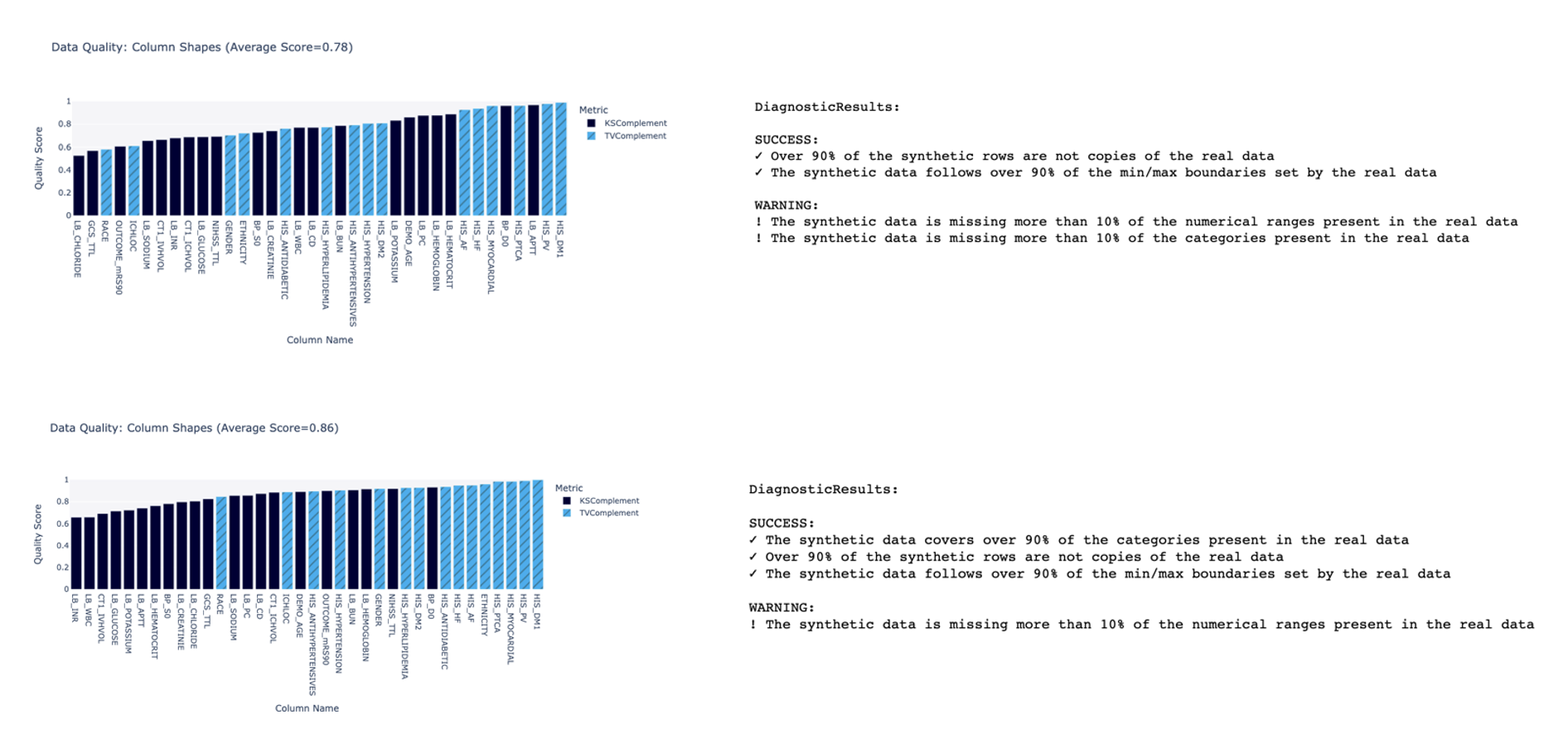

Supplement: S1 Fig — (PNG) [file pdig.0000493.s002.png]

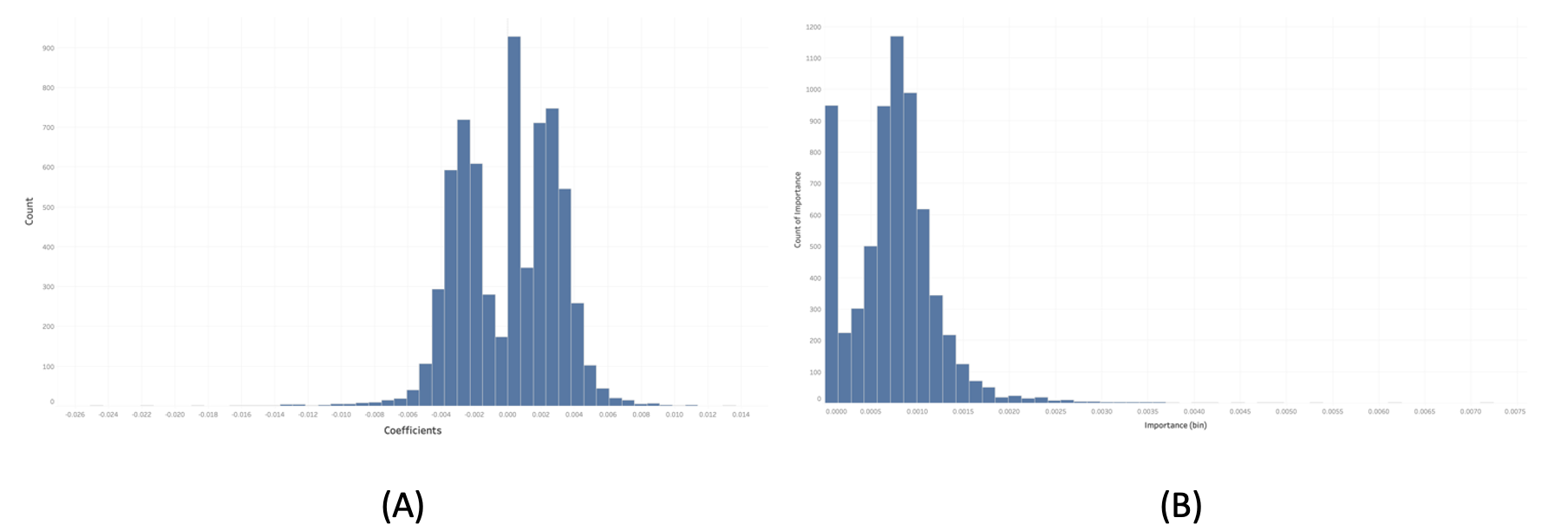

Supplement: S2 Fig — (PNG) [file pdig.0000493.s003.png]

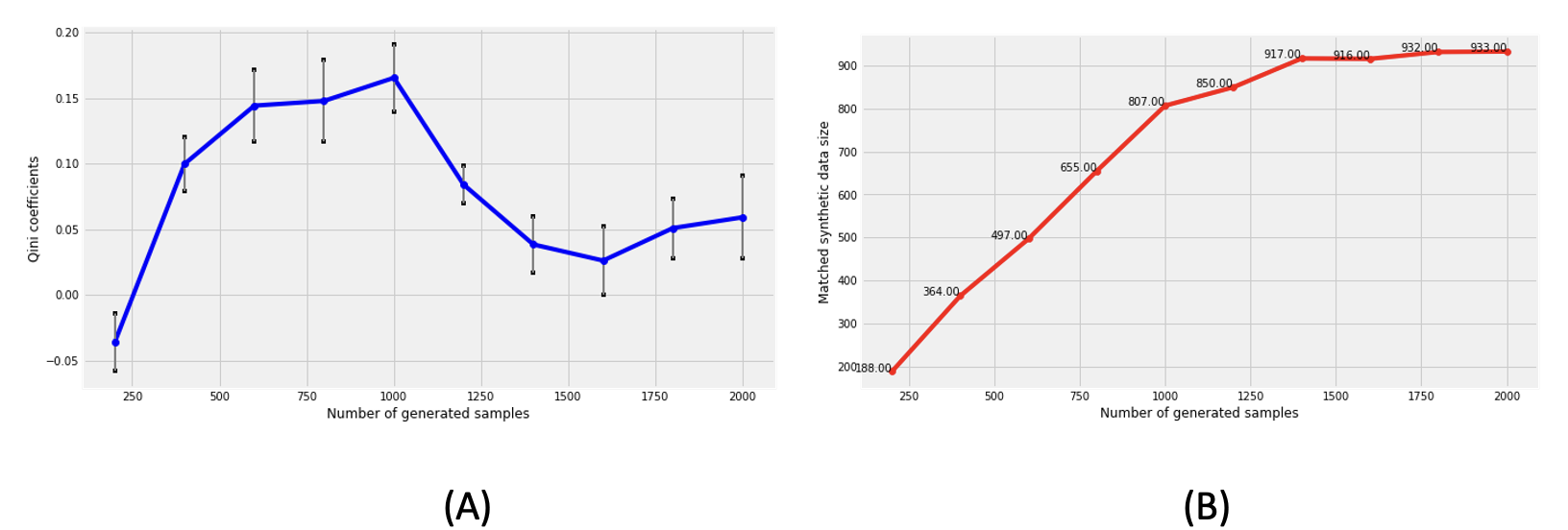

Supplement: S3 Fig — (PNG) [file pdig.0000493.s004.png]
