# Supplementary material for: An interpretable framework to identify responsive subgroups from clinical trials regarding treatment effects: Application to treatment of intracerebral hemorrhage
Source: PLOS Digit Health. 2024 May 7;3(5):e0000493. doi: 10.1371/journal.pdig.0000493 (PMC11075857; doi:10.1371/journal.pdig.0000493)
Supplement: S1 Table — The Qini-coefficient of the model is 0.1271. (DOCX) [file pdig.0000493.s005.docx]

**Supplementary Table 1.** Top 5 important rules/features that increase the treatment effects and top 5 rules/features that decrease the treatment effects of intensive blood pressure reduction therapy (ranked by importance score); NNT represents the total number of data points that fall in this subgroup in the training set; Subgroup ATE is estimated on all the ATACH2 data.

| **Subgroup Description** | **Coefficient** | **NNT (Support, %)** | **Importance** |
| --- | --- | --- | --- |
| **Favorable treatment effects** | | | |
| MAP<153.3 and DBP$\geq$70.0 | 0.0072 | 1287 (80.99%) | 0.0028 |
| Creatinine<0.9 and DBP$\geq$70.0 | 0.0041 | 703 (44.24%) | 0.0020 |
| Hyperlipidemia and GCS score<14 | 0.0070 | 116 (7.30%) | 0.0018 |
| NIHSS$\geq$3.0 and MAP<131.3 | 0.0043 | 1310 (82.44%) | 0.0016 |
| Potassium$\geq$5.4 | 0.0071 | 82 (5.16%) | 0.0016 |
| **Negative treatment effects** | | | |
| NIHSS<3.0 | -0.0097 | 148 (9.31%) | 0.0028 |
| DBP<70.0 and MAP<153.3 | -0.0081 | 141 (8.87%) | 0.0023 |
| DBP$\geq$145.0 | -0.0082 | 81 (5.10%) | 0.0018 |
| DBP<71 | -0.0053 | 149 (9.38%) | 0.0015 |
| NIHSS<3.0 and Potassium<3.7 | -0.0051 | 137 (8.62%) | 0.0014 |
